# Impaired Neurofilament Integrity and Neuronal Morphology in Different Models of Focal Cerebral Ischemia and Human Stroke Tissue

**DOI:** 10.3389/fncel.2018.00161

**Published:** 2018-06-18

**Authors:** Bianca Mages, Susanne Aleithe, Stephan Altmann, Alexandra Blietz, Björn Nitzsche, Henryk Barthel, Anja K. E. Horn, Constance Hobusch, Wolfgang Härtig, Martin Krueger, Dominik Michalski

**Affiliations:** ^1^Department of Neurology, University of Leipzig, Leipzig, Germany; ^2^Paul Flechsig Institute for Brain Research, University of Leipzig, Leipzig, Germany; ^3^Institute of Anatomy, University of Leipzig, Leipzig, Germany; ^4^Department of Nuclear Medicine, University of Leipzig, Leipzig, Germany; ^5^Institute of Anatomy, Histology and Embryology, Faculty of Veterinary Medicine, University of Leipzig, Leipzig, Germany; ^6^Institute of Anatomy and Cell Biology I and German Center for Vertigo and Balance Disorders, Ludwig Maximilian University of Munich, Munich, Germany

**Keywords:** cerebral ischemia, stroke, MCAO, neurofilaments, α-internexin, axonal spheroids

## Abstract

As part of the neuronal cytoskeleton, neurofilaments are involved in maintaining cellular integrity. In the setting of ischemic stroke, the affection of the neurofilament network is considered to mediate the transition towards long-lasting tissue damage. Although peripheral levels of distinct neurofilament subunits are shown to correlate with the clinically observed severity of cerebral ischemia, neurofilaments have so far not been considered for neuroprotective approaches. Therefore, the present study systematically addresses ischemia-induced alterations of the neurofilament light (NF-L), medium (NF-M), and heavy (NF-H) subunits as well as of α-internexin (INA). For this purpose, we applied a multi-parametric approach including immunofluorescence labeling, western blotting, qRT-PCR and electron microscopy. Analyses comprised ischemia-affected tissue from three stroke models of middle cerebral artery occlusion (MCAO), including approaches of filament-based MCAO in mice, thromboembolic MCAO in rats, and electrosurgical MCAO in sheep, as well as human autoptic stroke tissue. As indicated by altered immunosignals, impairment of neurofilament subunits was consistently observed throughout the applied stroke models and in human tissue. Thereby, altered NF-L immunoreactivity was also found to reach penumbral areas, while protein analysis revealed consistent reductions for NF-L and INA in the ischemia-affected neocortex in mice. At the mRNA level, the ischemic neocortex and striatum exhibited reduced expressions of NF-L- and NF-H-associated genes, whereas an upregulation for *Ina* appeared in the striatum. Further, multiple fluorescence labeling of neurofilament proteins revealed spheroid and bead-like structural alterations in human and rodent tissue, correlating with a cellular edema and lost cytoskeletal order at the ultrastructural level. Thus, the consistent ischemia-induced affection of neurofilament subunits in animals and human tissue, as well as the involvement of potentially salvageable tissue qualify neurofilaments as promising targets for neuroprotective strategies. During ischemia formation, such approaches may focus on the maintenance of neurofilament integrity, and appear applicable as co-treatment to modern recanalizing strategies.

## Introduction

Ischemic stroke represents one of the leading causes of death world-wide and is estimated to be the fourth most important cause of increased disability-adjusted life-years by 2030 (Donnan et al., 2008), causing long-lasting disabilities and thereby contributing to a high socio-economic burden (Mozaffarian et al., 2016). Despite enormous efforts in preclinical and clinical research, stroke therapy is still limited to intravenous administration of recombinant tissue plasminogen activator (rtPA) and mechanical thrombectomy to re-establish cerebral blood flow after an acute vessel occlusion (Hacke et al., 2008; Berkhemer et al., 2015). Moreover, based on the narrow therapeutic time window and other restrictions due to potential complications, only a small percentage of patients are currently eligible for these treatments (Dirks et al., 2011; Albers et al., 2018). Therefore, the need for the development of novel therapeutic approaches is still evident. Considering the translational roadblock from bench to bedside, i.e., the repeatedly failed translation of promising experimental approaches into the clinical setting (O’Collins et al., 2006), a detailed understanding of pathophysiological changes is mandatory for the development of novel and supportive treatment options.

Based on a comprehensive perspective of stroke-related tissue damage as captured by the “neurovascular unit” (NVU) and the penumbra concept (Astrup et al., 1981; Zlokovic, 2005; del Zoppo, 2009), experimental research has substantially improved the understanding of stroke pathophysiology (Dirnagl et al., 1999; Dirnagl, 2012). However, the underlying mechanism for the transition from an acute to long-lasting tissue damage is still ill-defined. In this context, proteins of the neurofilament network are assumed to play a pivotal role in maintaining cellular integrity (Yuan et al., 2012). In the setting of stroke, this concept is supported by two recent studies reporting on a significant correlation between the cerebrospinal fluid (CSF) level of neurofilament proteins and the degree of clinical stroke severity as well as the amount of white matter lesions in humans (Jonsson et al., 2010; Hjalmarsson et al., 2014).

In general, neurofilaments are elastic and fibrous proteins, representing essential and mechanically resilient components of the neuronal cytoskeleton (Wagner et al., 2007). They are considered as a particularly stationary and metabolically stable network (Yuan et al., 2009), which is integrated into the various other elements of the cytoskeleton, such as microtubules and actin filaments. Thereby, neurofilaments are involved in maintaining neuronal shape and the regulation of the axonal caliber (Friede and Samorajski, 1970; Hoffman et al., 1984, 1987). Here, they are shown to impact on conduction velocity and excitability and to facilitate the slow component of axonal transport (Griffin and Watson, 1988). The neurofilament protein of the central nervous system consists of three subunits: Neurofilament light (NF-L), neurofilament medium (NF-M) and neurofilament heavy (NF-H), which are named according to their molecular weights of 67–69 kD, 145-160 kD and 200 kD, respectively. These subunits are often called the neurofilament triplet, which is closely associated with α-internexin (INA), the latter of which is considered as a fourth and functionally interdependent neurofilament subunit (Yuan et al., 2006, 2012).

Under pathophysiological conditions, these neurofilament subunits are known to increase or accumulate in some neurodegenerative diseases. Such accumulations can most distinctly be found within cell bodies and proximal axons in patients suffering from amyotrophic lateral sclerosis (Julien et al., 1995), but high levels of neurofilaments, especially phosphorylated neurofilaments, have also been described for Alzheimer’s disease (Cork et al., 1986) and Parkinson’s disease (Forno et al., 1986). Remarkably, these observations are in line with experimental data obtained from mice, in which increased serum and CSF levels of NF-L were found to correspond with the progression of neurodegenerative diseases (Lu et al., 2015; Bacioglu et al., 2016a,b). Due to an assumed release from damaged neurons, a significant increase of NF-L in the CSF was regularly detectable in patients suffering from cerebral infarction (Norgren et al., 2003; Hjalmarsson et al., 2014). Further, increased serum levels of phosphorylated NF-H were found to correlate with the clinical outcome of stroke patients (Singh et al., 2011) and with experimentally induced neuronal damage (Shaw et al., 2005).

Since critical affections of the neurofilament network are likely to represent a central mechanism during the transition towards an irreversible neuronal damage after ischemic stroke, these structures may constitute possible targets for neuroprotective therapies. Therefore, the present study was aimed to systematically investigate ischemia-mediated affections of individual neurofilament proteins. Taking into account translational aspects, this study comprises different stroke models of middle cerebral artery occlusion (MCAO) in three different species, as well as human autoptic stroke tissue.

## Materials and Methods

### Study Design

All animal experiments were performed according to the European Union Directive 2010/63/EU and the German guideline for care and use of laboratory animals after approval by local authorities (Regierungspräsidium Leipzig; reference numbers TVV 34/11 for rats, TVV 51/14 and TVV 02/17 for mice, TVV 56/15 for sheep). Reporting of animal experiments followed the ARRIVE guidelines (Kilkenny et al., 2010).

Adult male C57BL/6 mice with a mean weight of 25 g, provided by Charles River (Sulzfeld, Germany), underwent a filament-based approach of permanent right-sided MCAO (fMCAO). Adult male Wistar rats with a mean weight of 300 g, also provided by Charles River, underwent right-sided thromboembolic permanent MCAO (eMCAO). Sufficient stroke-induced affection was confirmed using the Menzies Score (Menzies et al., 1992), ranging from 0 (no neuronal deficit) to 4 (spontaneous contralateral circling). Here, mice and rats had to demonstrate at least a score of 2, serving as pre-defined study inclusion criterion. Mice and rats were sacrificed 24 h after ischemia induction. In total, the study included six rats and five mice for immunofluorescence microscopy-based analyses, five mice for quantitative analyses using western blot and qRT-PCR, as well as four mice for electron microscopy. The analyses also comprised tissue from six male adult sheep, which underwent surgically induced permanent MCAO (sMCAO) with a follow-up period of 2 weeks. The sheep tissue was provided by the Faculty for Veterinary Medicine, University of Leipzig, Germany.

Further data were obtained from post-mortem brain tissue of a patient suffering from ischemic stroke provided by J. Kattah (Department of Neurology, University of Illinois College of Medicine, Peoria, IL, United States). Autoptic brainstem sections comprised ischemia-affected areas of the lateral medulla of a 61-year-old male individual who died 3 weeks after onset of stroke. Further details on the analyzed brain tissue and stroke characterization are given in Kattah et al. (2017).

### Induction of Focal Cerebral Ischemia in Mice, Rats, and Sheep

In mice, fMCAO was performed according to Longa et al. (1989), with minor modifications as previously described (Hawkes et al., 2013). In brief, mice were deeply anesthetized by intraperitoneal application of etomidate (33 mg/kg body weight, Hypnomidate, Janssen-Cilag, Neuss, Germany) followed by insertion of a standardized silicon-coated 6-0 monofilament (Doccol Corporation, Redlands, CA, United States) into the internal carotid artery. The filament was carefully pushed forward to the origin of the right middle cerebral artery until bending was observed or resistance felt.

In rats, eMCAO was applied according to Zhang et al. (1997), with slight modifications as described before (Michalski et al., 2009). In brief, anesthesia was achieved using 2–2.5% isoflurane (Baxter, Unterschleißheim, Germany) and a vaporizer (VIP 3000, Matrix, New York, NY, United States) providing a mixture of 70% N_2_O/30% O_2_. A PE-50 catheter with a previously prepared weight-adapted blood clot was positioned in the distal section of the internal carotid artery after insertion via the external carotid artery. Here, the blood clot was injected with a small bolus of saline followed by careful catheter removal. During surgery, the body temperature of mice and rats was controlled and adjusted to 37°C with a rectal probe and a thermostatically regulated warming pad (Fine Science Tools, Heidelberg, Germany).

In sheep, sMCAO was performed as described previously by Nitzsche et al. (2016). Briefly, sheep were anesthetized by an intravenous injection of ketamine (4 mg/kg body weight; Ketamin, Medistar, Holzwicke, Germany), xylazine (0.1 mg/kg body weight; Xylazin, Ceva Sante Animal GmbH, Düsseldorf, Germany), and diazepam (0.2 mg/kg body weight; Temmler Pharma GmbH, Marburg, Germany). During surgery, anesthesia was maintained by mechanical ventilation with 2% isoflurane and 40% oxygen (Primus, Dräger AG, Lübeck, Germany). The left temporal bone was exposed, followed by trepanation with a 6 mm barrel burr at 10,000 rpm (microspeed uni, scil animal care company, Viernheim, Germany). Next, the dura mater was incised and the proximal middle cerebral artery was occluded by electrosurgical coagulation using neurosurgical bipolar forceps (ME 411, KLS Martin, Tuttlingen, Germany). Thereafter, the dura mater was repositioned followed by suturing of muscles and skin. Finally, the animals were treated with antibiotics (enrofloxacin, 5% Baytril, Bayer AG, Leverkusen, Germany) and analgesics (Butorphanol, Alvegesic 1%; CP-pharm, Burgdorf, Germany) and were allowed to wake up after surgery. The presence of cerebral infarctions was confirmed by PET/MR imaging (Werner et al., 2015).

### Tissue Preparation

Mice and rats used for immunofluorescence microscopy were sacrificed followed by transcardial perfusion with saline and 2.5–4% paraformaldehyde (PFA) in phosphate buffered saline (PBS). After removal from the skull, the brains were post-fixed in the same fixative for 24 h, followed by equilibration in 30% phosphate-buffered sucrose. For fluorescence labeling, the forebrains were serially cut into coronal 30 μm-thick sections using a freezing microtome (Leica SM 2000R, Leica Biosystems, Wetzlar, Germany). All rodent brain sections were then stored at 4°C in 0.1 M Tris-buffered saline, pH 7.4 (TBS), containing 0.2% sodium azide. Mice used for western blot and qRT PCR analyses were sacrificed and perfused with saline only. After removal from the skull, brains were manually cut and ischemia-affected areas, being demarked by the ischemia-associated edema, as well as the respective contralateral regions were dissected, snap-frozen in liquid nitrogen and stored at -80°C.

Sheep were euthanized, brains were removed, and coronal specimens were obtained. Subsequently, the about 10 mm-thick equally spaced slices were immersed into 4% buffered PFA for 14 days. The slices were then equilibrated in 30% phosphate-buffered sucrose and consecutively cut at 40 μm thickness using a freezing microtome (Microm HM 430, Thermo Fisher Scientific, Waltham, MA, United States). All specimens were collected in 0.1 M TBS, pH 7.4, containing 0.2% sodium azide and stored at 4°C.

Human tissue was cut into 5 mm slices followed by formaldehyde fixation and embedding in paraffin. The tissue was further sectioned and mounted onto microscope slides. Prior to immunofluorescence labeling, paraffin sections were deparaffinized in xylene and rehydrated in graded alcohols, followed by antigen retrieval in 0.1 M citrate buffer, pH 6, for 15 min in a microwave oven at 96°C.

### Immunofluorescence Labeling and Microscopy

Prior to immunofluorescence labeling, the tissue was thoroughly rinsed in TBS and incubated in TBS blocking buffer containing 5% normal donkey serum and 0.3% Triton X-100 for 1 h. Next, primary antibodies (**Table 1**) were added for overnight incubation. Sections were washed with TBS followed by incubation with mixtures of appropriate fluorochromated secondary immunoreagents (**Table 1**) in TBS containing 2% bovine serum albumin for 1 h at room temperature. For the successive labeling of rabbit-anti-INA and biotinylated rabbit-anti-NF-L, the remaining free binding sites of secondary antibodies were blocked with TBS buffered 50% normal rabbit serum for 4 h, followed by overnight incubation of biotinylated-rabbit-anti-NF-L and visualization with Cy3-streptavidin for 1 h. All sections were extensively rinsed with TBS. Animal sections were mounted onto fluorescence-free microscope slides, dried, and cover-slipped with Entellan^®^ in toluene (Merck, Darmstadt, Germany). Human brain tissue was further treated with Sudan Black B (Merck) to quench tissue-specific autofluorescence (Schnell et al., 1999) and cover-slipped with glycerol gelatin (Sigma, Taufkirchen, Germany). For all the applied experiments, the omission of primary antibodies resulted in the absence of any labeling. Images for qualitative and quantitative data were captured with the Biorevo BZ-9000 microscope (Keyence, Neu-Isenburg, Germany), whereas high-magnification images were acquired with a confocal laser-scanning microscope (LSM 510 Meta, Carl Zeiss Microscopy, Göttingen, Germany).

**Table 1:** Immunoreagents used for immunohistochemistry.

Immunoreagent	Dilution	Manufacturer
**Primary antibody**		
Rabbit-anti-neurofilament L	1:200	Synaptic Systems, Göttingen, Germany
Biotinylated rabbit-anti-neurofilament L IgG	1:100	Synaptic Systems
Rabbit-anti-α-internexin (clone EPR1529)	1:500	Abcam, Cambridge, United Kingdom
Guinea pig-anti-neurofilament M	1:200	Synaptic Systems
Guinea pig-anti-neurofilament H	1:200	Synaptic Systems
Biotinylated mouse-anti-neurofilament H IgG (clone NE14)	1:100	Merck Millipore, Billerica, MA, United States
Mouse-anti-MAP2 (clone HM-2)	1:500	Sigma, Taufkirchen, Germany
Mouse-anti-HSP70 (clone C92F3A-5)	1:200	Enzo, Lausen, Switzerland
Guinea pig-anti-NeuN	1:200	Synaptic Systems
**Secondary antibody/immunoreagent**		
Cy2-donkey-anti-rabbit IgG	20 μg/ml each	Dianova (Hamburg, Germany) as supplier for Jackson ImmunoResearch (West Grove, PA, United States)
Cy3-donkey-anti-rabbit IgG		
Cy5-donkey-anti-guinea pig IgG		
AlexaFluor488-donkey-anti -mouse IgG		
Cy2-streptavidin		
Cy3-streptavidin		

### Fluorescence Intensity Measurements

For semi-quantification of NF-L-, NF-M-, NF-H- and INA-related fluorescence intensity in the model of fMCAO in mice, regions of interest (ROIs) were defined in the ischemia-affected areas of the neocortex and the striatum and equally mirrored to the contralateral hemisphere using the BZ-II-Viewer Software (Keyence). In detail, three consecutive forebrain slices per animal and per area with a distance of 300 μm apart were selected with the middle slice demonstrating the most pronounced ischemic lesion. For neocortical areas, 8 ROIs with a field dimension of 181 μm × 136 μm and a distance of 72 μm between their boundaries were distributed within the neocortical layers II-IV of the somatosensory cortex and parietal cortex. ROIs were defined from medial to lateral, extending from the unaffected to the most affected tissue as indicated by NF-L labeling. In the striatum, 12 ROIs with a field dimension of 362 μm × 272 μm and a distance of 144 μm between their boundaries were evenly distributed within the area of the striatum. Here, 4 ROIs each were placed in the central, transitional, and peripheral area of increased NF-L-related immunoreactivity. All ROIs were mirrored to the contralateral, non-affected hemisphere and served as controls. In summary, 24 cortical ROIs and 36 striatal ROIs were captured per hemisphere, immunolabeling and animal (*n* = 5). Micrographs were captured using a 12-bit CCD camera (Keyence) at a constant exposure time for each immunolabeling and area (striatum and neocortex). Prior to intensity measurements, a minimum threshold for each immunolabeling was set in order to minimize the error caused by unspecific background intensities. Thresholds had been adjusted individually for each immunolabeling until the background intensity could be nearly excluded without affecting or deleting specific signals. The fluorescence intensity of each ROI was measured using ImageJ software (National Institutes of Health, Bethesda, MD, United States) by analyzing the integrated density, which reflects the mean gray value per analyzed area.

### Western Blot and Quantitative Real-Time PCR (qRT-PCR)

For western blot analyses each brain sample was homogenized and lysed by ultrasonification in 60 mM Tris–HCl, pH 6.8, containing 2% sodium dodecyl sulfate (SDS) and 10% sucrose, supplemented with a protease inhibitor cocktail (Cell Signaling, Leiden, The Netherlands) on ice, followed by centrifugation at 13,000 rpm and 4°C for 10 min. Protein concentrations were then measured using the BCA kit (Thermo Fisher, Waltham, MA, United States). Next, proteins were denaturated in sample buffer (250 mM Tris–HCl, pH 6.8, containing 4% SDS, 10% glycerol, and 2% β-mercaptoethanol) at 95°C for 5 min. Proteins were separated using a 12.5% SDS-PAGE and transferred to nitrocellulose membranes (Th.Geyer, Renningen, Germany). Then, membranes were blocked with 5% dry milk in TBS (50 mM Tris–HCl, 150 mM NaCl, pH 7.5) for 30 min and incubated with primary antibodies (**Table 2**) at 4°C overnight. After three rinsing steps with washing buffer (6 g/l Tris, 8.8 g/l NaCl, 3 ml/l Tween 20), membranes were incubated with horseradish peroxidase-conjugated secondary antibodies (**Table 2**) for 1 h and developed with the ECL kit (Thermo Fisher). After image acquisition, membranes were stripped with stripping buffer (15 g/l glycine, 1 g/l SDS, 10 ml/l Tween 20, pH 2.2) and reused to detect β-actin as housekeeping protein for reference. The relative protein concentration of NF-L and INA was calculated from the respective β-actin immunosignal.

**Table 2:** Antibodies used for western blot.

Immunoreagent	Dilution	Manufacturer
**Primary antibody**		
Mouse-anti-neurofilament L (clone DA2)	1:1000	Thermo Fisher, Waltham, MA, United States
Rabbit-anti-neurofilament L	1:2000	Synaptic Systems, Göttingen, Germany
Rabbit-anti-α-internexin	1:5000	Abcam, Cambridge, United Kingdom
Mouse-anti-β-actin	1:2000	Cell Signaling, Danvers, MA, United States
**Secondary antibody**		
HRP-horse-anti-mouse IgG	1:10000	Vector Laboratories, Burlingame, CA, United States
HRP-goat-anti-rabbit IgG	1:10000	Vector Laboratories

For qRT-PCR, the total RNA was extracted according to the peqGOLD RNAPure^TM^ manual (VWR, Darmstadt, Germany) and RNA concentration was quantified by using a NanoDrop spectrophotometer (VWR). From each sample 500 ng RNA was reversely transcribed into cDNA using the ProtoScript M-MuLV First Strand cDNA Synthesis Kit (New England Biolabs Inc., Ipswich, MA, United States) and a mixture of oligo(dT) and random primers according to the manufacturer’s instructions. For qRT-PCR measurements, the levels of mRNA transcripts were quantified by duplication using the Rotor-Gene Q Instrument and the conformed Rotor-Gene SYBR Green PCR Kit (Qiagen, Hilden, Germany) according to the manufacturer’s instructions. For this purpose, the correlated gene-specific primer pairs (**Table 3**) were previously designed with the online tool Primer3web version 4.1.0^1^ and produced by biomers.net GmbH (Ulm, Germany). The mRNA levels were measured using the relative standard curve analysis, in which each sample was tested in triplicate and *Actb* was used as endogenous control gene (Saksi et al., 2011).

**Table 3:** Primer used for PCR.

Target gene	Encoded protein	Forward primer	Reverse primer
*Nefl*	NF-L	TCAAGGCTAAGACCCTGGAG	AGGCCATCTTGACATTGAGG
*Ina*	INA	AAATGGCCCTTGACATTGAG	TGGGAGGGAGCAAATAACTG
*Nefm*	NF-M	AAACTCCTAGAGGGGGAAGA	GCCTCGACTTTGGTCTTCTG
*Nefh*	NF-H	ACTCTCAGAGGCAGCCAAAG	AGCAGGTCCTGGTACTCTCG
*Actb*	β-actin	CATCCGTAAAGACCTCTATGCCAAC	ATGGAGCCACCGATCCACA

### Electron Microscopy

For clear-cut identification of ischemia-affected areas, fluorescein isothiocyanate (FITC)-conjugated albumin was intravenously applied 1 h prior to sacrifice to demark areas with impaired blood-brain barrier (BBB) integrity. Animals were sacrificed and transcardially perfused with saline and a fixative containing 4% PFA and 0.5% glutaraldehyde. Then, brains were removed from the skull and post-fixed in the same fixative for 24 h. Next, the brains were coronally cut into serial sections with a thickness of 50 μm using a vibratome (Leica Microsystems, Wetzlar, Germany) in cooled PBS. Ischemia-affected areas with impaired BBB were identified using peroxidase-conjugated anti-FITC IgG (1:2000 = 0.5 μg/ml; Dianova, Hamburg, Germany) and diaminobenzidine as previously described (Krueger et al., 2017). Sections were then further stained with 0.5% osmium tetroxide (EMS, Hatfield, PA, United States) for 30 min, followed by subsequent rinsing in PBS and dehydration with 30, 50, and 70% ethanol (J. T. Baker, Deventer, The Netherlands). After application of 1% uranyl acetate (Serva) in 70% ethanol for 1 h, the sections were further dehydrated using 80%, 90%, 96%, 100% ethanol and propylene oxide (Sigma-Aldrich, Steinheim, Germany). The sections were incubated in Durcupan (Sigma-Aldrich) and embedded between coated microscope slides and cover slips prior to the polymerization process at 56°C for 48 h. Areas showing BBB breakdown were identified by light microscopy, marked, and transferred onto blocks of resin for a second polymerization step. After trimming of the blocks, ultra-thin sections with a thickness of 55 nm were cut using an ultra-microtome (Leica Microsystems). Finally, the sections were transferred on formvar-coated grids and stained with lead citrate for 6 min. Ultrastructural analysis was performed using a Zeiss SIGMA electron microscope (Zeiss NTS, Oberkochen, Germany).

### Statistical Analyses and Image Processing

The acquired data was processed with Graph Pad Prism 5.01v (GraphPad Software Inc., La Jolla, CA, United States). Since ischemia-affected areas were compared with corresponding contralateral control areas, the Wilcoxon test was applied to check for statistical significance. In general, data are given as means ± standard error of mean (SEM) and a value of *p* < 0.05 was considered statistically significant. Figure panels were generated with Microsoft PowerPoint (version 2015; Microsoft Corp., Redmond, WA, United States). If necessary, brightness and contrast of images were slightly adjusted without creating or deleting signals.

## Results

### Consistently Altered Immunoreactivities of Neurofilament Proteins Throughout the Applied Animal Models of MCAO

**FIGURE 1:**
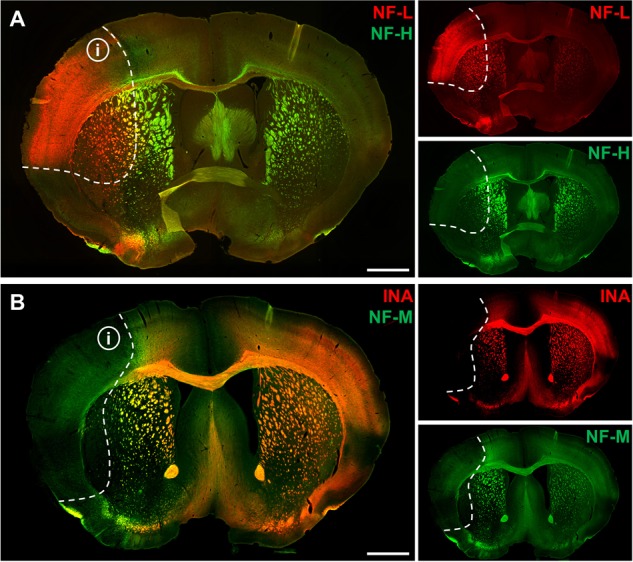
Affection of neurofilament subunits in mice 24 h after experimental stroke induction. Representative overview showing double immunofluorescence labeling of NF-L and NF-H **(A)** as well as of INA and NF-M **(B)** 24 h after fMCAO. Ischemia-affected areas are demarcated by an increase of NF-L immunoreactivity, while the immunosignals for INA, NF-M, and NF-H are found to be decreased. Dashed lines outline the border of increased NF-L immunoreactivity within ischemia-affected areas (i). Scale bars: 1 mm.

**FIGURE 2:**
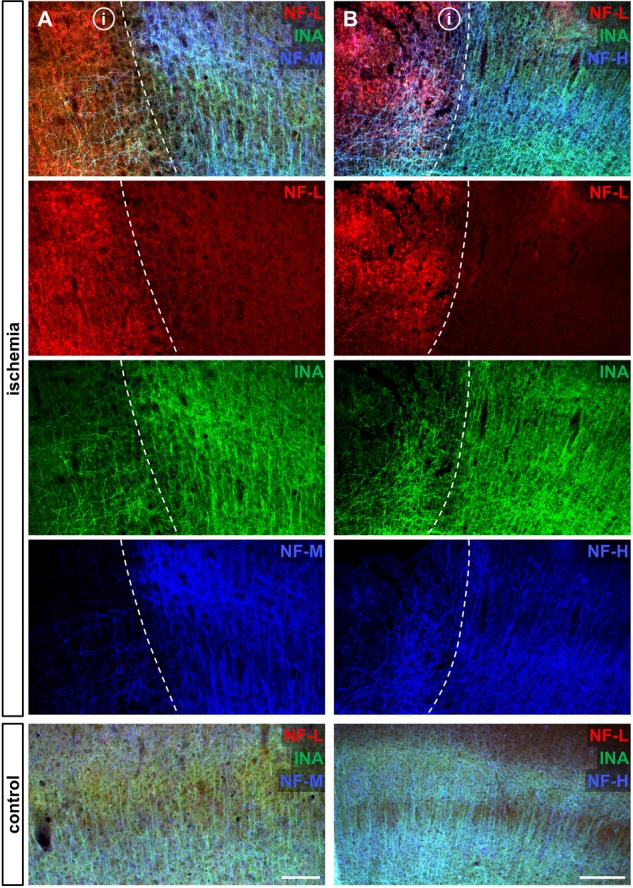
Opposite alterations of neurofilament subunits at the infarct border after fMCAO in mice. Triple immunofluorescence labeling of NF-L, INA, and NF-M **(A)** as well as of NF-L, INA, and NF-H **(B)** is shown at the ischemic border zone and in respective contralateral control areas in the neocortex of mice 24 h after ischemia induction. Notably, NF-L appears to be upregulated in the ischemia-affected area, whereas the same area is characterized by decreased immunoreactivities of INA, NF-M, and slightly of NF-H. Dashed lines outline the border of increased NF-L immunoreactivity within ischemia-affected areas (i). Scale bars: **(A)** 100 μm; **(B)** 200 μm.

**FIGURE 3:**
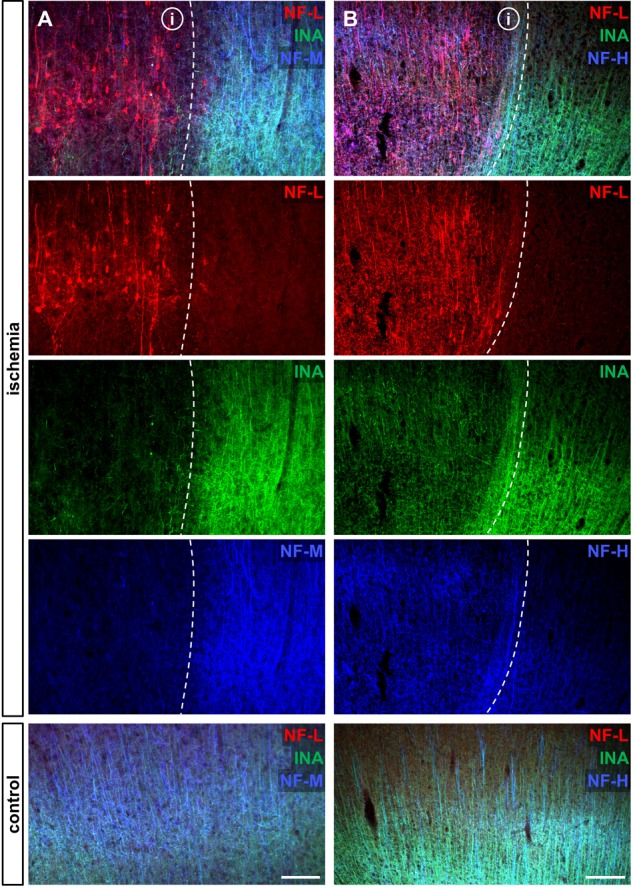
Opposite alterations of neurofilament subunits at the infarct border after eMCAO in rats. Triple immunofluorescence labeling of NF-L, INA, and NF-M **(A)** as well as of NF-L, INA, and NF-H **(B)** is shown at the ischemic border zone and in respective contralateral control areas in the model of eMCAO in rats 24 h after ischemia induction. Notably, in the ischemia-affected area NF-L appears to be upregulated, whereas the same area is characterized by decreased immunoreactivities of INA and NF-M. While the reduction of NF-H related immunolabeling appears to be faint, structural deformations of fibers and cellular processes became visible **(B)**. Dashed lines outline the border of increased NF-L immunoreactivity within ischemia-affected areas (i). Scale bars: **(A)** 100 μm; **(B)** 200 μm.

To address the expression patterns of NF-L, NF-M, NF-H, and INA in ischemia-affected tissue, we applied double immunofluorescence labeling for NF-L and NF-H as well as NF-M and INA in the mouse model of fMCAO and the rat model of eMCAO at 24 h after ischemia induction. Here, severe alterations of the respective expression patterns were observed in neocortical as well as striatal areas, when compared to the contralateral, unaffected hemisphere (**Figure 1**). While the immunoreactivities for INA, NF-M, and NF-H appeared to be decreased in ischemia-affected regions, an increased fluorescence intensity was observed for NF-L. As the most prominent differences were observed for INA and NF-L, immunofluorescence labeling of these proteins was combined with labeling of NF-M or NF-H. Thus, analyses of the infarct border consistently revealed decreased fluorescence intensities for INA and NF-M, while the immunoreactivity of NF-L was found to be increased in the ischemic areas throughout the models of fMCAO (**Figure 2**) and eMCAO (**Figure 3**). In contrast, rather inconsistent changes of the immunosignal were observed for NF-H. However, especially in the model of eMCAO, structural deformations of fibers and aberrant cellular processes became apparent by NF-H labeling in border zones of the ischemic lesion (**Figure 3**). These observations were confirmed in sheep after sMCAO, with most pronounced changes observed for NF-L and INA (Supplementary Figure S1), while NF-H immunoreactivity remained unaltered (data not shown).

### Increased NF-L Immunoreactivity Extends to the Ischemic Penumbra

Further, we tried to determine whether or not the described alterations include areas of potentially salvageable tissue according to the concept of an infarct core and a shell-like surrounding penumbra (Astrup et al., 1981). Therefore, we applied double immunofluorescence labeling of NF-L and microtubule-associated-protein-2 (MAP2), since the attenuation of MAP2 labeling is described to include penumbral areas of cerebral ischemia (Dawson and Hallenbeck, 1996; Kharlamov et al., 2009; Popp et al., 2009; Härtig et al., 2016). Further, according to Sharp et al. (2000) the outer layers of the ischemia-affected penumbra can be identified by an increased immunosignal of heatshock protein 70 (HSP70), since the expression of HSP70 is considered to represent an endogenous protective mechanism that occurs in neurons of the penumbra but not of the ischemic core (Kato et al., 1995; Weinstein et al., 2004). Therefore, triple immunofluorescence labeling of NF-L in combination with HSP70 and neuronal nuclei (NeuN) was applied.

**FIGURE 4:**
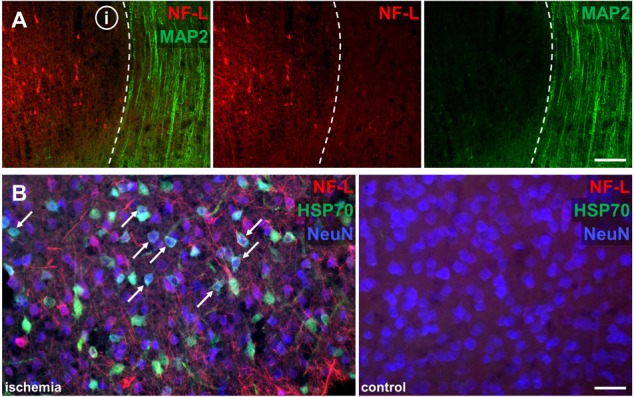
Co-labeling of NF-L with ischemia-sensitive neuronal markers Double immunofluorescence labeling illustrates the alternate affection of NF-L and MAP2-related immunoreactivity. The border of the ischemic area (i) is characterized by a clear-cut loss of MAP2-related immunolabeling (dashed line), whereas the immunoreactivity of NF-L is increased **(A)**. The ischemic penumbra is demarked by upregulation of HSP70 in NeuN-positive neurons (white arrows), which co-localizes with upregulated NF-L immunoreactivity, demonstrating ischemia-derived affection of NF-L in potentially salvageable tissue **(B)**. The corresponding contralateral area serves as control. Scale bars: **(A)** 100 μm; **(B)** 75 μm.

Of note, ischemia-related loss of MAP2 labeling co-localized with a clear-cut enhancement of the NF-L-related immunoreactivity (**Figure 4A**). Further, the increased immunoreactivity of NF-L also comprised areas of HSP70-positive neurons (**Figure 4B**), thus representing penumbral areas.

### Semi-Quantitative Analyses of Neurofilament-Related Immunoreactivity

**FIGURE 5:**
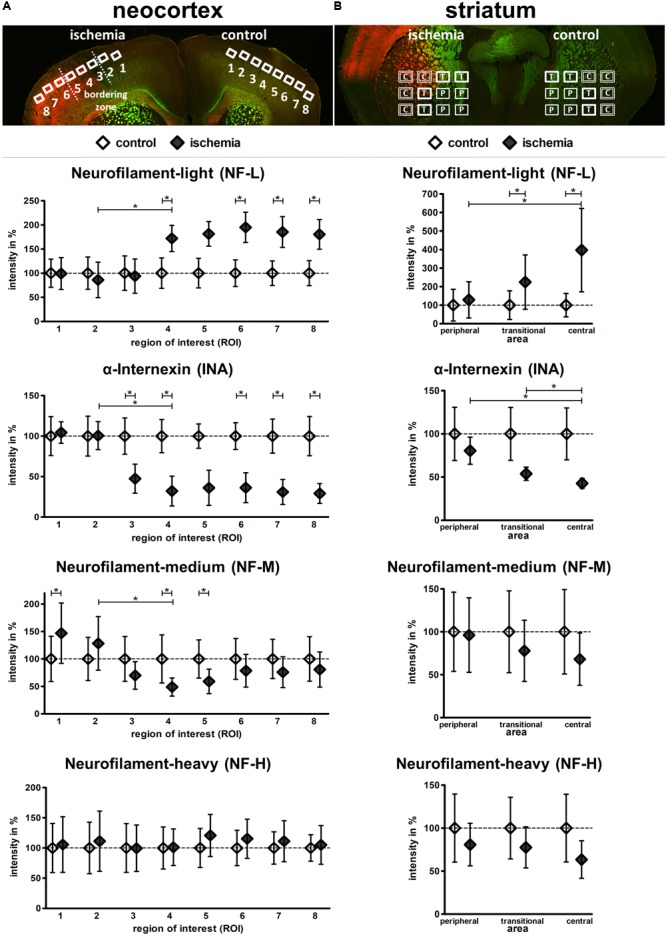
Semi-quantification of NF-L, INA, NF-M, and NF-H immunofluorescence intensity in the model of fMCAO. Coronal mouse brain sections labeled with NF-L (red) and NF-H (green) illustrate the predefined ROIs in the cortex **(A)**, as well as the peripheral (P), transitional (T), and central (C) areas of the striatum **(B)**. Semi-quantification reveals a significant increase of NF-L immunoreactivity, as well as a significant decrease of INA immunoreactivity in the ischemic tissue of the neocortex **(A)** and striatum **(B)**. NF-M immunoreactivity is significantly decreased in the ischemic neocortex, while NF-H immunoreactivity is not significantly altered **(A)**. NF-M and NF-H show a trend towards decreased immunoreactivities in the ischemic striatum **(B)**. Data are given as mean values; error bars indicate SEM. ^∗^*p* < 0.05; *n* = 5.

To quantify the described ischemia-related alterations, the individual fluorescence intensity for each of the investigated neurofilament components was measured in the model of fMCAO and compared with control areas (**Figure 5**). Thereby, the fluorescence intensity of NF-L was found to significantly increase in ischemic cortical areas compared to the contralateral hemisphere (ROI 4–8: 171.9–180.3%) as well as from ROI 2 to 4, representing the border zone towards the ischemia-affected tissue. This increase was similarly confirmed in the ischemic striatum from peripheral to central areas as well as in direct comparison to the contralateral regions (central: 396.8%; transitional: 224.4%).

In contrast to NF-L and in a rather opposite expression pattern, the fluorescence intensity of INA (**Figure 5**) was shown to be significantly reduced when compared to the contralateral hemisphere (ROI 3–8: 47.4–29.1%) and explicitly from ROI 2 to 4, representing the border zone towards the ischemia-affected tissue. This decrease of INA immunoreactivity was further evident from peripheral to central areas in the ischemia-affected striatum (transitional: 53.8%; central: 42.7%). In addition, a similar but less pronounced decrease was observed for NF-M (**Figure 5**). Here, a significant decrease became evident at the border zone of the ischemic tissue (ROI 2–4) and in ROI 4 (48.9%) and 5 (59%) compared to the contralateral hemisphere. In striatal areas, a relative decrease of NF-M fluorescence intensity was observed, but failed to reach statistical significance. In line with the less pronounced alterations of NF-H-related fluorescence intensity, its semi-quantitative analysis did not reveal significant differences (**Figure 5**).

### Western Blot Reveals Reduced NF-L and INA Protein Levels in Ischemia-Affected Tissue

**FIGURE 6:**
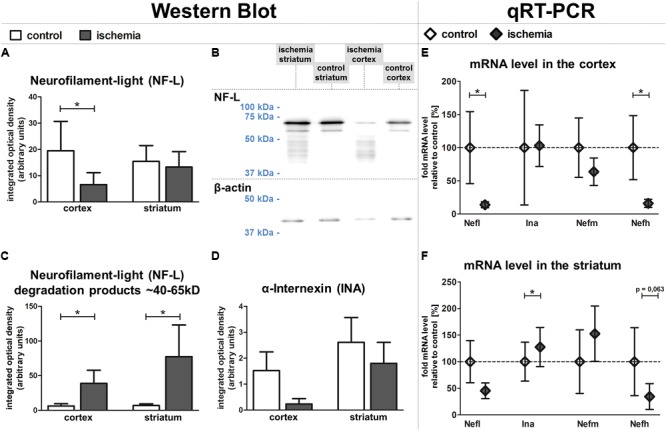
Western blot and qRT-PCR analyses of fMCAO tissue. Western blot analyses reveal a significantly lower protein level of NF-L in the ischemic neocortex and a slightly lower level in the ischemic striatum **(A,B)**. The ischemia-affected striatum and neocortex exhibit increased bands of lower molecular weight (∼40–65 kDa) compared to control areas **(B,C)**. Western blot analysis reveals a trend towards decreased INA expression in the ischemic neocortex compared to control areas **(D)**. qRT-PCR shows significantly decreased mRNA levels of *Nefl* and *Nefh* in the cortex **(E)**, while the mRNA level of *Ina* is significantly increased in the striatum **(F)**. *Actb* served as endogenous control gene **(E,F)**. Bars and squares indicate mean values; error bars indicate SEM. ^∗^*p* < 0.05; *n* = 5.

To rule out that the observed increase of NF-L-related immunoreactivity is caused by better accessibility of the antibody in the ischemia-affected and structurally impaired tissue, we further explored relative protein levels of NF-L and additionally INA using western blot analysis. Here, the protein amount of NF-L was found to be significantly reduced in ischemic neocortical areas when compared to the contralateral hemisphere, whereas in the striatum, this trend failed to reach statistical significance (**Figures 6A,B**). However, western blot analysis revealed an increase of detectable bands with associated lower molecular weight in ischemic regions (**Figure 6C**), which are known to relate to NF-L degradation products (Posmantur et al., 1994). Thereby, a strong and significant increase of bands ranging from 40–65 kDa was observed in neocortical and striatal areas when compared to the contralateral regions (**Figure 6C**). Since the decreased levels of the 68 kDa NF-L protein appear to be contradictory to the increased immunofluorescence intensity observed in histological sections, additional western blot analyses were performed using another established monoclonal mouse-anti-NF-L antibody (DA2) with proven specificity in ischemia-affected tissue (Schroeder et al., 2003). In line with the aforementioned findings (**Figure 6A**), the NF-L (DA2)-detected protein amount in the ischemic neocortex and striatum was also reduced in comparison to the contralateral hemisphere (Supplementary Figures S2a,b), but failed to reach statistical significance. Importantly, the NF-L degradation products smaller than 65 kDa were not detected by the monoclonal mouse-anti-NF-L antibody (DA2) in the western blot (Supplementary Figure S2b), which corresponds with decreased fluorescence intensities in the ischemia-affected tissue, thereby co-localizing with the increased immunosignal of the rabbit NF-L antibody (Supplementary Figure S2c).

In conformity with the data obtained from immunofluorescence microscopy, protein levels of INA appeared to be reduced with the most pronounced ischemia-related affections in neocortical areas (**Figure 6D**). Taken together, western blot analyses revealed an ischemia-induced relative reduction of NF-L and INA protein levels, which predominantly affected neocortical regions.

### Reduced *Nefl* and *Nefh* mRNA Levels in Combination With an Upregulation of *Ina* in Ischemic Areas

In addition to analyses at the protein level, we further investigated the mRNA levels of *Nefl* (NF-L), *Ina* (INA), *Nefm* (NF-M), and *Nefh* (NF-H) using qRT-PCR (**Figures 6E,F**). The mRNA level of *Nefl* was significantly decreased in ischemia-affected neocortical areas, whereas in the striatum the differences did not reach statistical significance. In contrast, the mRNA level of *Ina* is shown to be significantly upregulated in the striatum, but not in the neocortex. Further analyses addressing the mRNA level did not reveal significant inter-hemispheric differences for *Nefm*, but a significant downregulation for *Nefh* in the ischemic neocortex.

### Comparable Alterations of Neurofilaments in Human Stroke Tissue

**FIGURE 7:**
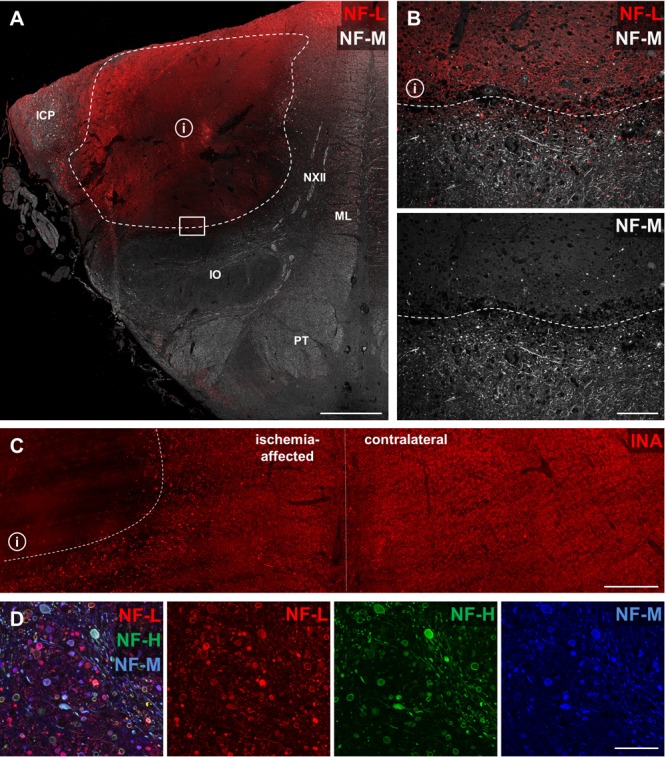
Opposite affections of neurofilament subunits and morphological alterations in human post-mortem stroke tissue. Overview of human autoptic brain stem tissue with histopathologically verified infarct (i) delineated by a dashed line **(A)**. Higher magnification reveals alterations comparable to the findings in animal MCAO models with most profound ischemia-derived affection of NF-L and INA. NF-L shows an increased immunoreactivity within the ischemic area **(B)**, while NF-M **(B)** and INA **(C)** appear to be decreased. A representative image illustrates characteristic morphological bead-like neuronal alterations, which can be visualized using each of the applied neuronal markers at high magnification **(D)**. Dashed lines indicate ischemia-affected areas. Rectangle in **(A)** illustrates the area captured in **(B)** at higher magnification. Abbreviations: ICP, inferior cerebellar peduncle; NXII, hypoglossal nerve; ML, medial lemniscus; IO, inferior olive; PT, pyramidal tract. Scale bars: **(A)** 2 mm; **(B)** 200 μm; **(C)** 750 μm; **(D)** 100 μm.

To address the question of whether the described alterations of NF-L, NF-M, NF-H, and INA in the applied stroke models also hold true for the human pathophysiology, we performed respective immunofluorescence labeling on human autoptic stroke tissue. Importantly, human stroke tissue exhibited a comparable enhancement of NF-L-related immunoreactivity as previously described in the animal models (**Figure 7A**). Higher magnification reveals a clear-cut border of the increased NF-L immunosignal (**Figure 7B**, dashed line) which correlates with the previously characterized ischemic border zone (dashed line, **Figure 7A**) of the analyzed brain stem tissue (Kattah et al., 2017). In addition and in line with the animal models, decreased immunoreactivities were observed for NF-M (**Figure 7B**) and INA (**Figure 7C**) in ischemia-affected areas. In line with the animal models, the decrease of NF-H-related fluorescence intensity appeared much less pronounced compared to NF-M and INA (not shown). Notably, NF-L, NF-M, and NF-H labeling regularly revealed characteristic morphological alterations, captured by higher magnification (**Figure 7D**). Here, spheroid axonal structures, as signs of axonal damage (Kattah et al., 2017), were regularly found in ischemia-affected tissue, especially in affected white matter fiber tracts (**Figure 7D**).

### Characteristic Morphological Alterations in Stroke Models and Human Autoptic Stroke Tissue

**FIGURE 8:**
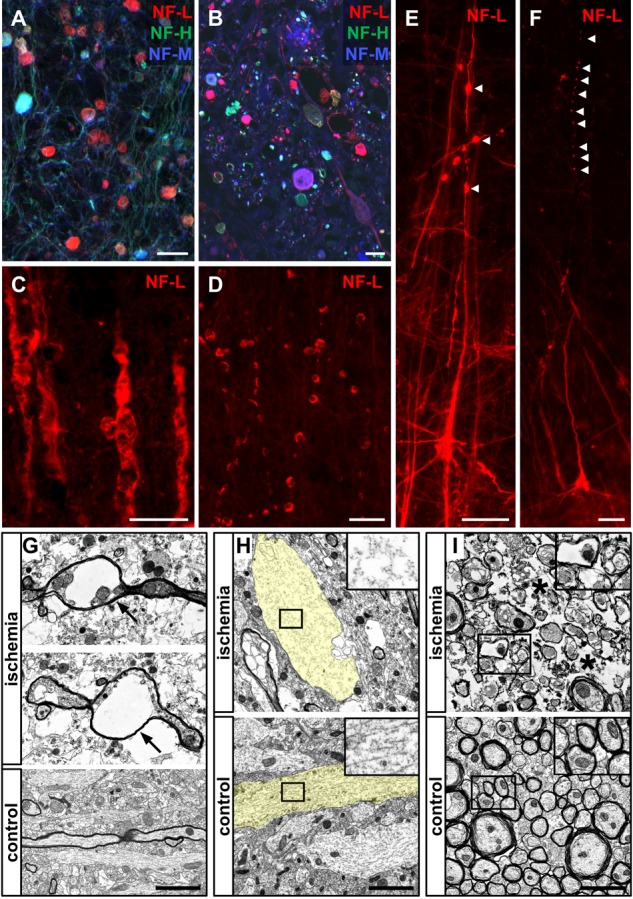
Morphological alterations of neuronal processes due to ischemia demonstrated by immunofluorescence and electron microscopy. Morphological alterations as demarcated by NF-L, NF-M, and NF-H labeling observed in human stroke tissue **(A)** are detectable throughout the applied animal models of MCAO **(B–F)**. NF-L immunolabeling reveals swollen and morphologically altered axons **(C)** and bulb-like vacuolization of NF-L-positive structures **(D)**. Of note, bead-like deformations (arrow heads) are visible in continuity with neuronal processes **(E)** as well as apparently discontinuous to respective axons or dendrites (arrow heads) **(F)**. Electron microscopy images obtained from the neocortex in the model of fMCAO illustrate severe alterations of the neuronal cytoskeleton resulting in vacuolization of axons (arrows) **(G)** comparable to the bead-like structures captured by immunofluorescence microscopy **(D–F)**. Further, non-myelinated dendrites often appear less electron dense and swollen (transparently highlighted in yellow) indicative of a cellular edema, resulting in an impaired cytoskeletal order (insets) with lost alignment of microtubules and neurofilaments **(H)**. Swollen and structurally altered cellular processes with an impaired cytoskeleton are also observed in the ischemia-affected striatum **(I)**. Generally, a cellular edema is also detectable in surrounding ischemia affected glial cells (asterisks). Scale bars: **(A–F)** 25 μm; **(G–I)** 2 μm.

After detecting morphological alterations by visualization of neurofilament proteins in human ischemia-affected tissue (**Figure 8A**), higher magnification revealed similar structures indicative of axonal damage, such as spheroid and bead-like structures in all the animal models applied (**Figures 8B–F**). In line with the observations in human stroke tissue (**Figures 7D, 8A**), spheroid and bead-like structures are visualized by NF-L, NF-M, and NF-H labeling (**Figure 8B**). Further, high magnification revealed irregular, bulged, and swollen appearing neuronal processes (**Figure 8C**) as well as bead-like NF-L-positive structures which appeared to be discontinuous to adjacent neuronal structures (**Figure 8D**). Partly, the described bead-like formations appeared to be connected to neuronal processes, such as apical dendrites (**Figure 8E**), whereas in other cases, the integrity of the respective processes was found to be lost (**Figure 8F**).

### Structurally Impaired Axons and Dendrites Revealed by Electron Microscopy

To further analyze these alterations at an ultrastructural level, electron microscopy regularly revealed bead-like vacuolations of affected myelinated axons (**Figure 8G**). Moreover, captured dendrites often appeared to be swollen and less electron-dense, indicative of the ischemia-associated cellular edema (**Figure 8H**). In these cases, the regular alignment of cytoskeletal elements, such as microtubules and neurofilaments, is shown to be impaired (**Figure 8H**, inset). Importantly, the described alterations were not only observed throughout the cortical areas (**Figures 8G,H**), but were also present in the ischemia-affected striatum (**Figure 8I**). In general, the ischemia-associated cellular edema was found to not only affect neurons, but also surrounding glial processes (**Figures 8G–I**).

## Discussion

Although modern recanalizing therapies have proven beneficial in clinical trials (Hacke et al., 2008; Berkhemer et al., 2015), they are still limited to only about one fourth of acutely affected patients (Dirks et al., 2011). Furthermore, the translational roadblock from bench to bedside is illustrated by the remarkable number of more than 1,000 experimental treatment strategies, all of which failed successful translation into the clinical setting (O’Collins et al., 2006). Therefore, a detailed understanding of stroke pathophysiology is essential for successful development of neuroprotective therapies targeting the transition from acute to irreversible and long-lasting tissue damage. So far, neurofilament proteins proved to be sensitive markers of brain damage (Martínez-Morillo et al., 2015) and were further shown to correlate with the clinical outcome of stroke patients (Singh et al., 2011; Traenka et al., 2015). As ischemia-derived alterations of these neuronal elements might represent a central mechanism during the transition process towards long-lasting tissue damage (Härtig et al., 2016), the present study was aimed to systematically characterize ischemia-associated affections of the neurofilament network.

In this study, stroke-related alterations of the four critical subunits NF-L, NF-M, NF-H, and INA were for the first time analyzed by using a multi-parametric approach including immunofluorescence labeling, western blot, qRT-PCR as well as electron microscopy. Considering translational aspects, this study was designed according to the STAIR recommendations and thus includes three different animal models and species as well as human autoptic stroke tissue in order to enhance reproducibility and validity (Fisher et al., 2009).

Notably, in all MCAO models, immunoreactivities for INA, NF-M, and partly for NF-H were consistently found to be decreased, while NF-L immunofluorescence intensity related to the applied polyclonal rabbit-anti NF-L antibody appears clearly increased. Among the investigated proteins, especially NF-L and INA exhibited the most striking and significant ischemia-derived affections, showing a sharp border of an increased NF-L immunoreactivity co-localized with strikingly decreased immunoreactivities for INA.

Importantly, the described alterations of NF-L protein expression are not confined to areas of neuronal cell death in the infarct core (Astrup et al., 1981). Instead, we were able to demonstrate that the detectable increase of NF-L immunoreactivity not only delineates the ischemic border zone as outlined by the loss of MAP2 immunolabeling (Dawson and Hallenbeck, 1996; Härtig et al., 2016), but further co-localizes with areas exhibiting HSP70-positive neurons (**Figure 4**). In fact, this is of particular interest, as the area of neuronal HSP70 expression was previously described to exceed the area of ischemia-mediated neuronal cell death (Sharp et al., 2000; Weinstein et al., 2004). In these areas, HSP70 expressing neurons are presumed to survive the ischemic insult (Abe et al., 1992; Li et al., 1993; Klettner, 2004). Thus, the described alterations of the neuronal cytoskeleton comprise areas of the ischemic penumbra (Kato et al., 1995), which represents potentially salvageable tissue (Sharp et al., 2000), thereby distinguishing neurofilaments as potential targets for future neuroprotective strategies.

However, the ischemia-derived increase of NF-L immunoreactivity is rather contradictory to previous reports, which demonstrate reduced immunoreactivities for neurofilaments in histological sections and western blot analysis after traumatic brain injury and stroke (Ogata et al., 1989; Inuzuka et al., 1990; Posmantur et al., 1994; Schroeder et al., 2003). For this reason, western blot analysis of NF-L and INA was added. Importantly, this method revealed reduced levels for both the 68 kDa NF-L protein and INA (**Figures 6A,D**). However, western blot analysis revealed that the polyclonal rabbit-anti-NF-L antibody used for immunohistochemistry not only detects the 68 kDa NF-L protein, but also degradation products smaller than 68 kDa (**Figures 6B,C**) as described by Posmantur et al. (1994). This significant increase of detectable NF-L degradation products in the ischemic tissue might well explain the enhanced NF-L immunofluorescence intensity in histological sections with the applied polyclonal rabbit-anti-NF-L antibody (**Figure 5**). Of note, decreased protein levels of NF-L were further confirmed using a monoclonal mouse-anti-NF-L (DA2) antibody (Supplementary Figure S2), which does not recognize NF-L degradation products and therefore provides decreased immunofluorescence intensities on histological sections. This decrease precisely co-localizes with increased NF-L immunosignals related to the polyclonal rabbit-anti-NF-L antibody (Supplementary Figure S2c).

Although the general use of the monoclonal mouse-anti-NF-L antibody may seem more appropriate as it shows decreased immunoreactivities in histological sections in line with decreased protein levels in the western blot, its use for stroke research may be impaired on widely applied unfixed or shortly fixed snap-frozen tissue. Here, secondary antibodies would also detect intrinsic unfixed IgG, thereby spoiling the intended analysis. Therefore, the applied polyclonal rabbit-anti-NF-L antibody proved to be a valuable tool to consistently detect alterations of NF-L throughout the applied animal models and human stroke tissue, and further reliably allows the detection of the ischemia-affected areas. These findings qualify the applied NF-L antibody as a highly ischemia-sensitive marker, whose sensitivity compared to NF-M and NF-H can be explained by its higher susceptibility to proteolytic degradation (Petzold, 2005), as well as the stoichiometry of the subunit composition of 5:3:1 for NF-L:NF-M:NF-H (Lobsiger and Cleveland, 2009), which renders NF-L immunolabeling proportionally more susceptible to altered protein levels.

In order to systematically characterize cytoskeletal alterations in the analyzed tissue, efforts were made to supplement data on the respective mRNA levels. The reduced mRNA levels for *Nefl* and *Nefh*, might reflect the incapacity of affected cells to maintain their functioning cytoskeleton (**Figure 6E**) and are in line with previous reports demonstrating a reduced NF-H protein expression 3 days after ischemia-induction in a model of MCAO (Inuzuka et al., 1990). Although a robust reduction of NF-H immunofluorescence intensity was not observed in the present study, these changes are likely to be preceded by the reduced *Nefh* mRNA level (**Figures 6E,F**) since the half-lives of neurofilament proteins appear to be much longer compared to the respective mRNA (Barry et al., 2007; Maier et al., 2009). On the other hand, the protein level of INA was reduced in striatal regions, whereas the *Ina* mRNA level appeared to be increased (**Figures 6D–F**). However, as mRNA levels usually exhibit a poor correlation with the respective protein levels (Maier et al., 2009) the time course of the described alterations further needs to be addressed by longitudinal studies including shorter and longer time points after ischemia induction.

Qualitatively, by applying immunofluorescence labeling of NF-L, NF-M, and NF-H bead-like and spheroid deformations of neuronal processes were observed (**Figures 7D, 8B**) showing axonal spheroids as a sign of axonal damage (Kattah et al., 2017). Such bead-like deformations have been reported to occur as axonal “blebbing” in cell culture experiments as soon as 15 min after the insult and are shown to lead to extensive axonal degeneration (Castillo and Babson, 1998). Accordingly, swelling of neurites was observed in the setting of focal cerebral ischemia (Uzdensky et al., 2016). Of note, in the present study, these bulb-like structures often appeared to be disconnected from each other as exemplified by NF-L labeling (**Figures 8D,F**). Similar observations were previously interpreted as a stage of bulb-like axonal disconnections (Dewar and Dawson, 1997). In the present study, we were able to identify morphological correlates for the observed spheroid-shaped alterations at an ultrastructural level. In detail, bead-like deformations were regularly observed for axons and signs of an intracellular edema became apparent in unmyelinated dendrites, thereby resulting in a loss of the regular alignment of neurofilaments and microtubules (**Figure 8H**). Further structural affections of axons and dendrites, as well as the surrounding glia were identified in cortical and striatal areas and substantiate the alterations visualized by immunofluorescence microscopy.

Although the present study design is rather descriptive, we here provided a systematic, translational-oriented approach to investigate alterations of neurofilaments due to focal cerebral ischemia applying a variety of animal models and human stroke tissue. Nevertheless, as the presented quantitative data are based on a single time point (24 h after ischemia induction), future studies are needed to analyze cytoskeletal damage in ischemic areas and respective border zones in a longitudinal fashion, from the very early period after ischemia onset over days and weeks. The given time point, however, was chosen to allow detection of cellular alterations in a time window that appears realistic for future pharmacological intervention. This time window is further known to correlate with an increased BBB permeability that harbors the risk of hemorrhagic transformation and cerebral edema formation (Huang et al., 1999; Durukan et al., 2009; Pillai et al., 2009), both representing clinically relevant complications associated with poor functional outcome (Battey et al., 2014; Stokum et al., 2016).

In summary, the robust evidence for critical quantitative and structural alterations of the neurofilament network in ischemia-affected areas, including potentially salvageable tissue, distinguishes neurofilament subunits as possible targets for upcoming therapeutic approaches. Ideally, a pharmacological modulation which optimizes cellular integrity by stabilizing the neurofilament network appears conceivable for both scenarios: First, as a novel neuroprotective strategy in a time window of at least 24 h, and second, as potential co-treatment in addition to modern techniques of vascular recanalization.

## Author Contributions

DM, WH, and MK contributed to the study design. DM, MK, BM, HB, and BN conducted the animal experiments. BM, MK, and DM prepared the manuscript. DM and MK contributed to the regulatory affairs on animal experiments. AH contributed to the histopathological evaluation and provision of human tissue. WH, BM, SuA, StA, and AB contributed to the immunohistochemistry and labeling. BM and SuA carried out the immunofluorescence microscopy. BM and MK performed the electron microscopy. CH and BM did the western blot analysis. SuA contributed to the qRT-PCR. BM and MK carried out the statistical analyses. BM contributed to the figure generation.

## Conflict of Interest Statement

The authors declare that the research was conducted in the absence of any commercial or financial relationships that could be construed as a potential conflict of interest.

## Footnotes

1 http://primer3.ut.ee/
