# Supplementary figures and images for: Impaired Neurofilament Integrity and Neuronal Morphology in Different Models of Focal Cerebral Ischemia and Human Stroke Tissue
Source: Front Cell Neurosci. 2018 Jun 18;12:161. doi: 10.3389/fncel.2018.00161 (PMC6015914; doi:10.3389/fncel.2018.00161)

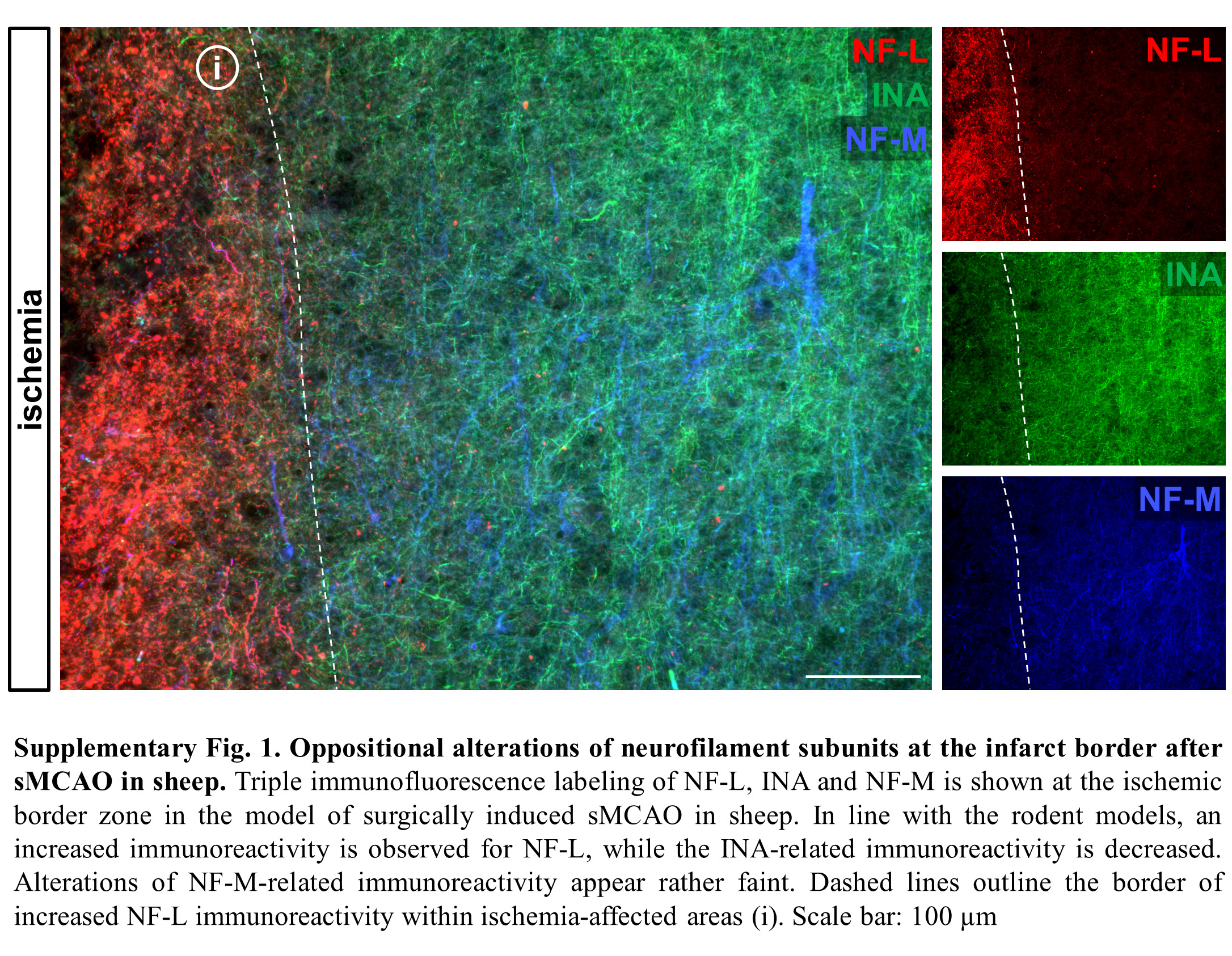

Supplement: Supplementary file 1 [file Image_1.jpg]

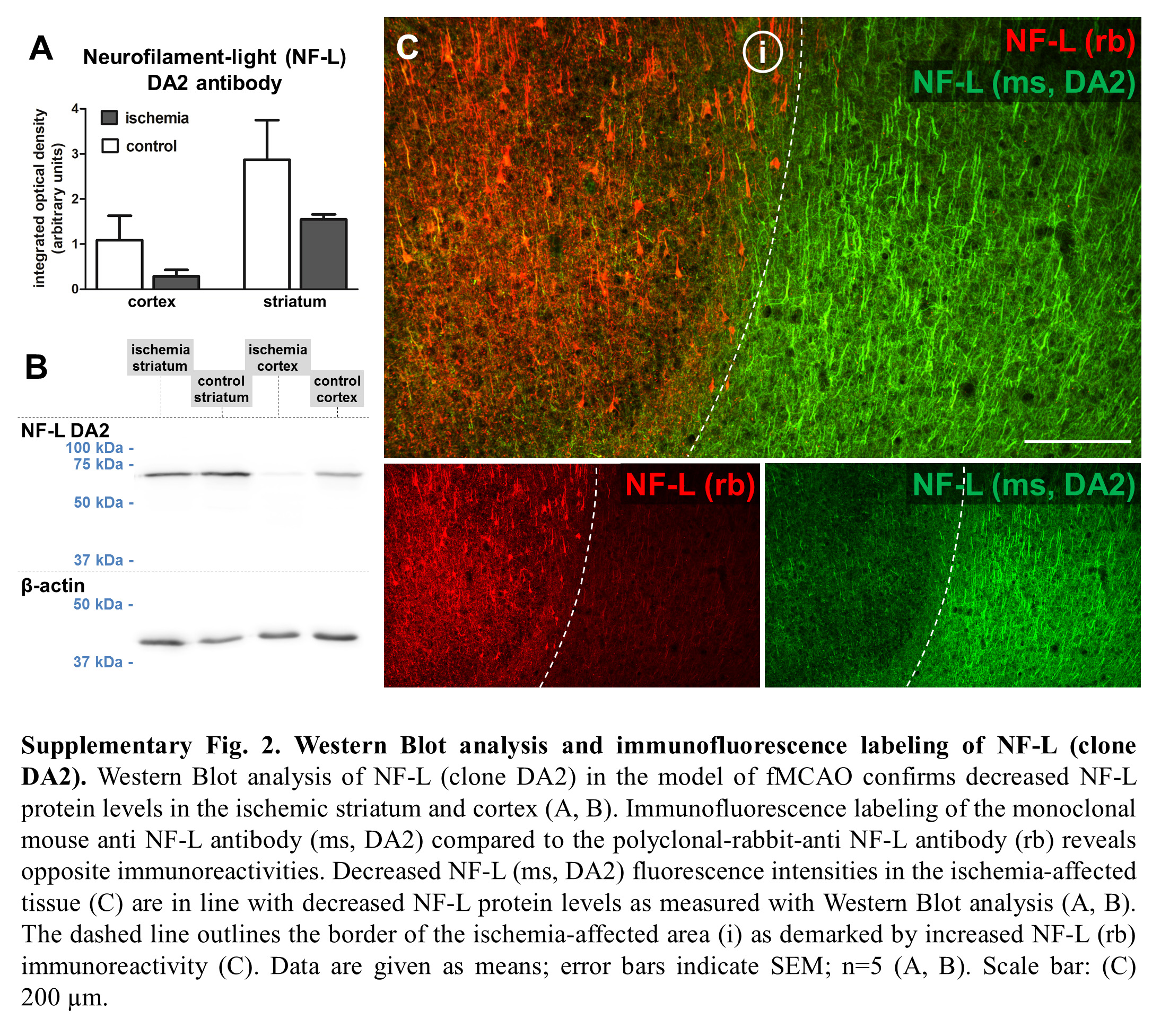

Supplement: Supplementary file 2 [file Image_2.jpg]
